# An Improved Method of Perineorrhaphy

**Published:** 1880-08

**Authors:** E. S. Bunker


### AN IMPROVED METHOD OF PERINEORRHAPHY.

E. S. BUNKER, M. D.

Laceration of the perineum, not involving the sphincter ani, is by no means a rare accident, and perineorrhaphy is much more frequently indicated than was formerly supposed. The operation has been greatly simplified in recent times, so that it belongs, not so much to the brilliant repertoire of the specialist, as to the every-day work of the general practitioner. The essential phases of the operation are three: 1, the denuding of the parts; 2, the adjustment of the sutures; 3, the removal of the sutures after union has taken place.

1. Denuding the parts.—The method which I employ is based upon a suggestion from my friend, Dr. John Merritt, of Brooklyn. With a scalpel I make a V-shaped incision through the integument. This marks the outer limits of the space that is to be denuded. Then, seizing the apex with a forceps or tenaculum, I dissect up the skin from the part to be denuded in a single triangular flap, which I leave attached by its base to the posterior wall of the vagina. This can be done with great rapidity, and there is absolutely no loss of tissue to the patient. The flap is then lifted out of the way by an assistant, and the sutures are inserted and secured in the ordinary way. It will now be found that the flap has become very much retracted from before backward, and that, from side to side, it is folded upon itself like a sheet thrown over a clothes-line. The apex now barely reaches to the ostium vaginæ, and the two lateral halves of the raw surface of the flap being brought into apposition quickly unite. The flap, treated in this way, roofs over the wounded parts beneath, and protects them from vaginal discharges. Ultimately it becomes a solid raphé, reinforcing the new-made perineal body.

2. The sutures.—Several years ago I published a note advocating the use of horse-hair sutures in perineorrhaphy. A subsequent experience in several scores of cases has not changed my estimate of their value. They are easy of adjustment, secure, easy of removal, and they give the patient relatively little pain while they are *in situ.* I use a needle curved in the segment of a circle, and introduce it by the aid of Sims' needle-holder. Two horse-hairs, doubled so as to make four strands, will always hold.

3. Removal of the sutures.—This I usually do about the seventh day. With horse-hair it is as a rule painless; with silver wire it is often attended with atrocious suffering.

This method of operating admits of great celerity. Two years ago I applied it in a case where nitrous oxide gas was administered by my friend, Dr. G. W. Brush, and the total duration of anæsthesia was only seventeen minutes, during which time every step of the operation was carried out. I have twice operated on lacerations through the sphincter, using only horse-hair sutures: the result in each case was satisfactory.—*Annals of Anatomical and Surgical Society, Brooklyn.*

